# Therapeutic effect of dienogest on adenosarcoma arising from endometriosis: a case report

**DOI:** 10.1186/2193-1801-2-618

**Published:** 2013-11-20

**Authors:** Nobutaka Tasaka, Koji Matsumoto, Toyomi Satoh, Takeo Minaguchi, Mamiko Onuki, Hiroyuki Ochi, Yumiko O Tanaka, Akiko Sakata, Masayuki Noguchi, Hiroyuki Yoshikawa

**Affiliations:** Department of Obstetrics and Gynecology, University of Tsukuba, 1-1-1 Tennodai, Tsukuba, 305-8575 Japan; Department of Radiology, University of Tsukuba, 1-1-1 Tennodai, Tsukuba, 305-8575 Japan; Depatment of Pathology, Faculty of Medicine, University of Tsukuba, 1-1-1 Tennodai, Tsukuba, 305-8575 Japan

**Keywords:** Dienogest, Progestin, Adenosarcoma, Endometriosis

## Abstract

Dienogest is a novel synthesized progestin used for treatment of endometriosis. This is the first case report describing a therapeutic effect of dienogest on a gynecologic malignancy. The patient was a 44-year-old woman with advanced adenosarcoma arising from the endometriosis in the rectovaginal space and infiltrating the left pelvic wall, left ureter, rectum and vagina. The residual tumor after tumor debulking surgery was resistant to both chemotherapy and radiotherapy. Dienogest was used as a substitute for medroxyprogesterone acetate because of the presence of deep vein thrombosis. Based on the RECIST criteria, partial response was obtained with oral dienogest therapy at six months and the serum CA125 level also decreased from 70 U/ml to 16 U/ml. The tumor remained stable up to 21 months. Thromboembolism or other adverse effects did not occur during the dienogest therapy. Dienogest may be useful for the treatment of adenosarcoma arising from endometriosis.

## Introduction

Müllerian adenosarcoma is a rare tumor characterized by a benign epithelial component and a sarcomatous stromal component (Clement et al. 1974; Clement et al. 1990). These tumors most commonly appear as lesions in the uterus, derived from the endometrium. To date, however, several cases of extrauterine adenosarcoma arising from a background of endometriosis have been reported (Hines et al. 2002; Liu et al. 2003; Raffaelli et al. 2004; Huang et al. 2009). Müllerian adenosarcoma is relatively insensitive to chemotherapy and radiation; thus, an optimal therapy for advanced or recurrent tumors remains to be defined. Histological finding of sarcomatous overgrowth is associated with a highly aggressive clinical behavior of the tumor.

Medroxyprogesterone acetate (MPA), a synthesized progestin, has a therapeutic effect on gynecological malignancies such as adenosarcoma (Hines et al. 2002; Lee et al. 2010), low-grade endometrial stromal sarcoma (Amant et al. 2009), and grade 1 endometrial carcinoma (Ushijima et al. 2007). However, MPA is not used for women that are at risk of thromboembolism because it is the most significant side-effect of MPA therapy (Kuhl et al. 2006).

We experienced a case of advanced adenosarcoma that was insensitive to either chemotherapy or radiotherapy. Since she had asymptomatic deep vein thrombosis, dienogest was used as a substitute for MPA for progesterone therapy.

### Case presentation

**Figure 1:**
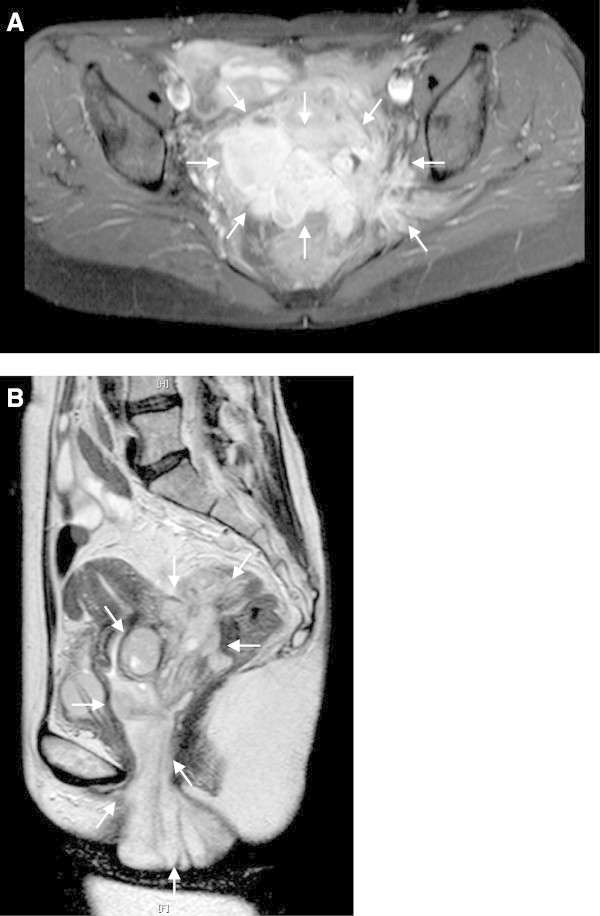
**MRI images before the primary surgery.** The tumor invaded the left pelvic wall (**A**; axial fat-saturated T1-weighted image) and the vagina (**B**; sagittal T2-weighted image).

The patient is a 44-year-old G2P2, who had a history of endometriosis. At 29 years old, she underwent laparoscopic surgery for endometriosis at a local hospital. At 40 years old, she was treated with GnRH analog for endometriosis at another facility. She developed a rapidly growing polypoid mass in the vagina and was referred to our hospital. Computed tomography (CT) and magnetic resonance imaging (MRI) showed a polymorphic mass in the pelvis. A major portion of the mass formed a 5-cm cystic lesion in the pouch of Douglas, infiltrating to the left acetabular, left ureter and rectum (Figure 1A). The remainder of the mass infiltrated through the posterior vaginal fornix, and formed the polypoid mass occupying the entire vagina (Figure 1B). She had no metastasis to lymph nodes or distant sites. The pathological analysis of the biopsy specimen revealed necrotic tissue. The CA125 was 336.5 U/ml and D-dimer (18.8μg/ml) was elevated. Doppler ultrasonography confirmed thrombosis of the right soleal vein. Chest CT demonstrated no evidence of pulmonary embolism. The patient received anticoagulant therapy with unfractionated heparin followed by warfarin. Thereafter, she was followed-up by D-dimer assay and Doppler ultrasonography. The polypoid mass necrosed and reformed during the hospital stay.

**Figure 2:**
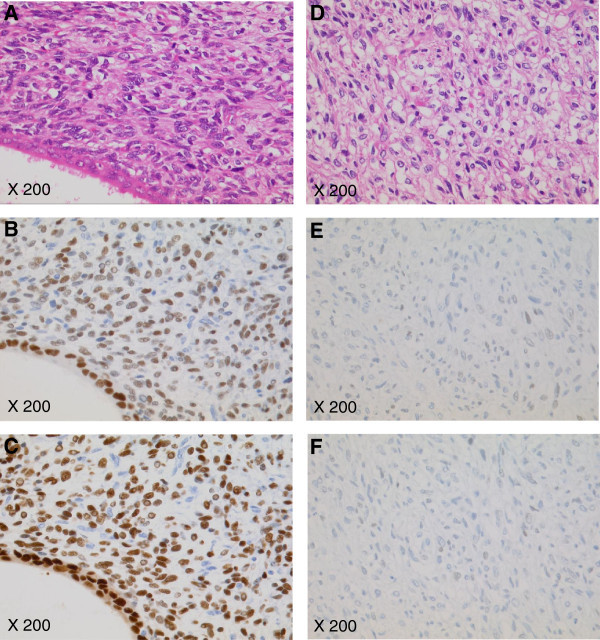
**Tumor Pathology.** At the primary surgery, the tumor was diagnosed as an adenosarcoma containing a benign epithelial component (an endometriotic gland, bottom left) surrounded by an atypical hypercellular stromal component (**A**; ×200). However, the autopsy following the rapid tumor regrowth revealed malignant stromal components with sarcomatous overgrowth (**D**; ×200). At autopsy, the expression levels of estrogen **(E)** and progesterone **(F)** receptors were reduced compared to those at the primary surgery (**B** and **C**, respectively).

**Figure 3:**
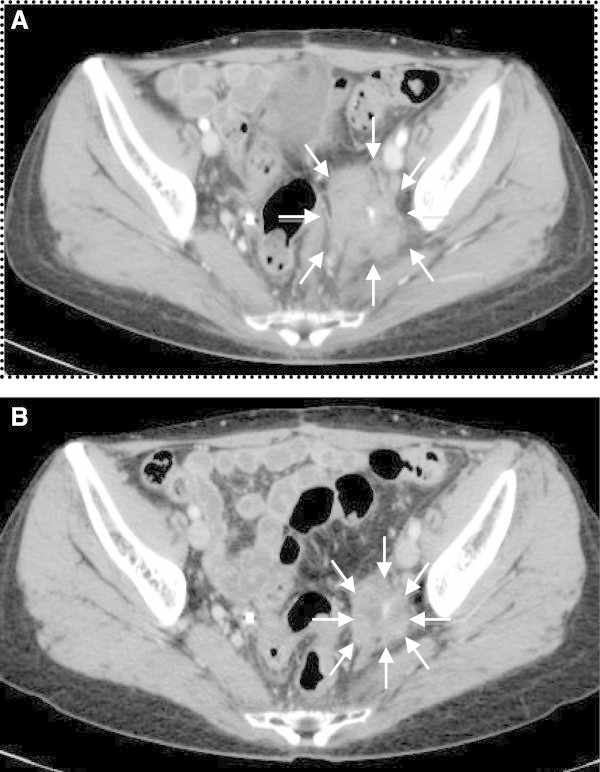
**CT images during dienogest therapy.** Based on the RECIST criteria, a partial response was obtained at 6 months after initiating oral DNG treatment (**A**: 0 months; **B**: 6 months) and the tumor was stable up to 21 months.

**Figure 4:**
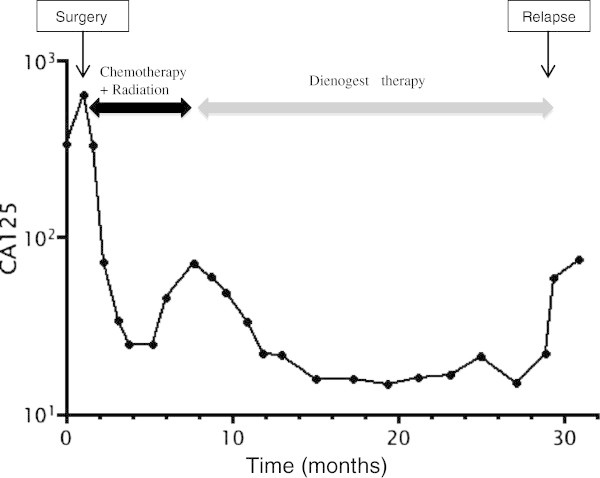
**CA125 changes through the clinical course.**

A total abdominal hysterectomy, bilateral salpingo-oophrectomy, omentectomy, and tumor debulking procedures were performed 1 month after the first medical examination. The uterus was normal-sized and the endometrium was intact; however, the posterior wall of the uterus was adherent to the pelvic mass. The left adnexa were partially involved to the mass. The right adnexa and the omentum appeared normal. Sub-optimal cytoreduction was achieved with the residual tumor remaining adherent to the left ureter, the rectum, and the left pelvic wall. She made an uneventful recovery and CA125 fell to 72.7 IU/ml immediately after the surgery. The final pathologic diagnosis was an extrauterine adenosarcoma arising from the recto-vaginal septum (Figure 2A). This tumor coexisted with endometriosis. The uterus contained normal endometrium without stromal atypia. Microscopically, the mitotic rate was 10-12 per 10 high-power fields. The epithelial component was positive for pankeratin and vimentin, but negative for α-SMA, CD10, and Ki-67. Both of the epithelial and stromal components were positive for estrogen and progesterone receptors (Figure 2B and 2C). Postoperatively, the patient underwent adjuvant chemotherapy with ifosmide and cisplatin (IFM: 1500mg/m^2^ day1, CDDP:20mg/m^2^ day1-day4; 4 week intervals). After three cycles of chemotherapy, an MRI revealed that the residual tumor appeared to be stable disease (SD) by RECIST. The CA125 was 24.7 IU/ml. Subsequently, she received cisplatin-based chemoradiotherapy (CCRT: total dose of 60 Gy irradiation to whole pelvis, CDDP 35mg/m^2^ weekly; 4 cycles). However, a follow-up MRI after CCRT demonstrated progressive disease by RECIST and the CA125 again rose to 70 U/ml. Therefore, the patient commenced oral DNG therapy at a dose of 2 mg daily. Written informed consent for DNG therapy was obtained from the patient. Based on the RECIST criteria, a partial response was obtained at six months after initiating oral DNG treatment (Figure 3). The CA125 gradually fell to 16 U/ml over a six month period (Figure 4) and no adverse effects occured. Although the tumor was stable for 21 months following dienogest therapy, she had a rapid tumor relapse 30 months after the surgery. The tumor regrew into a 10 cm mass, invading directly into the bladder and the rectum; she died from sepsis 1 month after the tumor relapse. The autopsy revealed a mixture of benign epithelial glands and malignant stromal components with sarcomatous overgrowth. In these stromal components, the expression levels of estrogen and progesterone receptors were reduced (Figure 2E and 2F).

## Discussion

Endometriosis has been reported to give rise to malignant transformation more frequently in ovarian (5.6%) than at extraovarian sites of endometriosis (1.6%) (Stern et al. 2001). In malignant tumors associated with extraovarian endometriosis, adenosarcoma is the second most common tumor after clear cell adenocarcinoma (Stern et al. 2001). In this case, adenocarcinoma appeared to arise from endometriosis in the rectovaginal space. The microscopic foci in the endometrium were found to be contiguous with the malignant stromal tissue and no other possible primary tumor site was found.

Progestins are usually used in the treatment of endometriosis, as well as for contraception and hormone replacement therapy. These compounds interact with progesterone receptors (PRs) to activate or repress gene expression in target cells. Synthetic progestines are structurally classified into two major chemical classes (Benagiano et al. 2004). Medroxyprogesterone acetate (MPA) is a 17β-hydroxyprogesterone derivative (C-21 progestogen) that is structurally related to progesterone, while norethisterone (NET) and levonorgestrel (LNG) are derivatives of 19-northesteron (C-19 nortestosterone) that are structurally related to testosterone. Of these compounds, it has been reported that MPA has a therapeutic effect on gynecological malignancies such as atypical endometrial hyperplasia (Ushijima et al. 2007), grade 1 endometrial carcinoma (Ushijima et al. 2007), low-grade endometrial stromal sarcoma (Amant et al. 2009), and adenosarcoma (Hines et al. 2002; Lee et al. 2010). However, thromboembolism is one of the most serious side-effects of MPA (Kuhl et al. 2006). Vein thromboembolism is found in 9.9% of women with endometrial cancer (Satoh et al. 2008) and 4.9% of those women with uterine sarcoma (Rodriguez et al. 2011). Thus, the use of MPA in the treatment of these malignancies may be limited.

Dienogest (DNG: 17α-cyanomethyl-17β-hydroxyestra-4,9-dien-3 one) is a novel ‘hybrid progestogen’ that has pharmacodynamic properties typical of the two main classes of progestogens (Ruan et al. 2012). DNG has antiovulatory activity that reduces serum estrogen level in vivo (Harada et al. 2009), and direct antiprolirative and anti-inflammatory effects on endometrial stromal cells in vitro (Okada et al. 2001). Therefore, DNG has a therapeutic effect on endometriosis (Harada et al. 2009). Although DNG has recently been launched for the treatment of endometriosis, DNG also has antiproliferative effects on estrogen receptosr (ERs)- and/or PR-positive endometrial and breast cancer cells in vitro, suggesting potential usefulness in the treatment of ER/PR-positive malignant tumors (Katsuki et al. 1997; Banno et al. 2012). DNG has considerable antiandrogenic activity; however, it has little effect on the metabolic and cardiovascular systems (Ruan et al. 2012). More importantly, DNG lacks glucocorticoid activity that can be associated with an increased risk of venous or arterial thromboembolism (Ruan et al. 2012; Herkert et al. 2001). To date, DNG-related thromboembolism has not been reported, even in long-term use (Momoeda et al. 2009) or high dose therapy (Schindler et al. 2010). Given its lack of hemostatic action, DNG appears to be more suitable for the treatment of endometrial cancer and uterine sarcoma than MPA because women with these tumors are at an increased risk of venous thrombosis (Satoh et al. 2008; Rodriguez et al. 2011).

In the presented case, the adenosarcoma underwent very rapid growth. In addition, this aggressive tumor was insensitive to either chemotherapy or radiotherapy. Several case reports have suggested that MPA may be effective in the treatment of adenosarcoma; however, MPA was contraindicated in the present case because she had deep vein thrombosis. Therefore, DNG was used as a substitute for MPA for progesterone therapy. A successful partial response was obtained at six months after initiating oral DNG treatment and the tumor was stable up to 21 months.

The rapid regrowth of the tumor following a long-term stable status may have been due to the reduction of ER and PR expression via histological evolution to sarcomatous overgrowth. In previous reports of adenosarcoma, successful treatment with MPA was demonstrated only for ER- and PR-positive tumors (Hines et al. 2002; Lee et al. 2010). Therefore, substantial levels of ER and PR expression may be necessary to induce the therapeutic effect of DNG, as well as MPA.

In conclusion, we experienced a case of advanced adenosarcoma that was treated with DNG. To the best of our knowledge, this is the first case report describing a therapeutic effect of DNG on a gynecologic malignancy. In this case, no adverse effects, including thromboembolism, were observed. DNG may be useful in the treatment of gynecologic malignancies, such as adenosarcoma, low-grade endometrial stromal sarcoma and grade 1 endometrial carcinoma, even for women who are at risk of thromboembolism.

### Consent

Oral informed consent was obtained from the patient for the publication of this report and any accompanying images before her death. Written informed consent was obtained from the family of the patient for the publication of this report and any accompanying images.

## References

[CR1] Amant F, Coosemans A, Debiec-Rychter M, Timmerman D, Vergote I (2009). Clinical management of uterine sarcomas. Lancet Oncol.

[CR2] Banno K, Kisu I, Yanokura M, Tsuji K, Masuda K, Ueki A, Kobayashi Y, Yamagami W, Nomura H, Susumu N, Aoki D (2012). Progestin therapy for endometrial cancer: the potential of fourth-generation progestin. Int J Oncol.

[CR3] Benagiano G, Primiero FM, Farris M (2004). Clinical profile of contraceptive progestins. Eur J Contracept Reprod Health Care.

[CR4] Clement PB, Scully RE (1974). Müllerian adenosarcoma of the uterus. A clinico- pathologic analysis of ten cases of a distinctive type of müllerian mixed tumor. Cancer.

[CR5] Clement PB, Scully RE (1990). Müllerian adenosarcoma of the uterus: a clinicopathologic analysis of 100 cases with a review of the literature. Hum Pathol.

[CR6] Harada T, Momoeda M, Taketani Y, Aso T, Fukunaga M, Hagino H, Terakawa N (2009). Dienogest is as effective as intranasal buserelin acetate for the relief of pain symptoms associated with endometriosis–a randomized, double-blind, multicenter, controlled trial. Fertil Steril.

[CR7] Herkert O, Kuhl H, Sandow J, Busse R, Schini-Kerth VB (2001). Sex steroids used in hormonal treatment increase vascular procoagulant activity by inducing thrombin receptor (PAR-1) expression: role of the glucocorticoid receptor. Circulation.

[CR8] Hines BJ, Porges RF, Mittal K, Muggia FM, Curtin JP (2002). Use of medroxyprogesterone acetate in the treatment of Müllerian adenosarcoma: a case report. Gynecol Oncol.

[CR9] Huang GS, Arend RC, Sakaris A, Hebert TM, Goldberg GL (2009). Extragenital adenosarcoma: a case report, review of the literature, and management discussion. Gynecol Oncol.

[CR10] Katsuki Y, Shibutani Y, Aoki D, Nozawa S (1997). Dienogest, a novel synthetic steroid, overcomes hormone-dependent cancer in a different manner than progestins. Cancer.

[CR11] Kuhl H, Stevenson J (2006). The effect of medroxyprogesterone acetate on estrogen-dependent risks and benefits: an attempt to interpret the Women's Health Initiative results. Gynecol Endocrinol.

[CR12] Lee SJ, Bae JH, Kim DC, Park JS, Namkoong SE (2010). Oral progesterone treatment in a young woman with müllerian adenosarcoma whose ovary was preserved: a case report. Int J Gynecol Cancer.

[CR13] Liu L, Davidson S, Singh M (2003). Müllerian adenosarcoma of vagina arising in persistent endometriosis: report of a case and review of the literature. Gynecol Oncol.

[CR14] Momoeda M, Harada T, Terakawa N, Aso T, Fukunaga M, Hagino H, Taketani Y (2009). Long-term use of dienogest for the treatment of endometriosis. J Obstet Gynaecol Res.

[CR15] Okada H, Nakajima T, Yoshimura T, Yasuda K, Kanzaki H (2001). The inhibitory effect of dienogest, a synthetic steroid, on the growth of human endometrial stromal cells in vitro. Mol Hum Reprod.

[CR16] Raffaelli R, Piazzola E, Zanconato G, Fedele L (2004). A rare case of extrauterine adenosarcoma arising in endometriosis of the rectovaginal septum. Fertil Steril.

[CR17] Rodriguez AO, Gonik AM, Zhou H, Leiserowitz GS, White RH (2011). Venous thromboembolism in uterine cancer. Int J Gynecol Cancer.

[CR18] Ruan X, Seeger H, Mueck AO (2012). The pharmacology of dienogest. Maturitas.

[CR19] Satoh T, Matsumoto K, Uno K, Sakurai M, Okada S, Onuki M, Minaguchi T, Tanaka YO, Homma S, Oki A, Yoshikawa H (2008). Silent venous thromboembolism before treatment in endometrial cancer and the risk factors. Br J Cancer.

[CR20] Schindler AE, Henkel A, Moore C, Oettel M (2010). Effect and safety of high-dose dienogest (20 mg/day) in the treatment of women with endometriosis. Arch Gynecol Obstet.

[CR21] Stern RC, Dash R, Bentley RC, Snyder MJ, Haney AF, Robboy SJ (2001). Malignancy in endometriosis: frequency and comparison of ovarian and extraovarian types. Int J Gynecol Pathol.

[CR22] Ushijima K, Yahata H, Yoshikawa H, Konishi I, Yasugi T, Saito T, Nakanishi T, Sasaki H, Saji F, Iwasaka T, Hatae M, Kodama S, Saito T, Terakawa N, Yaegashi N, Hiura M, Sakamoto A, Tsuda H, Fukunaga M, Kamura T (2007). Multicenter phase II study of fertility-sparing treatment with medroxyprogesterone acetate for endometrial carcinoma and atypical hyperplasia in young women. J Clin Oncol.

